# Expression of noggin, an antagonist of bone morphogenetic protein, in schwannoma: A possible mechanism

**DOI:** 10.3892/ol.2014.2138

**Published:** 2014-05-12

**Authors:** KEIKO KANEKO, CHIKAHISA HIGUCHI, NORIFUMI NAKA, HIDEKI YOSHIKAWA

**Affiliations:** Department of Orthopedic Surgery, Osaka University Graduate School of Medicine, Suita, Osaka 565-0871, Japan

**Keywords:** schwannoma, bone scalloping, noggin, bone morphogenetic protein

## Abstract

Schwannoma is a benign peripheral nerve sheath tumor derived from Schwann cells. Bone scalloping, including spinal foramen enlargement, develops when the tumor is located adjacent to a bone and is a characteristic radiological feature. In the present study, to investigate the pathomechanism of bone resorption, the expression of noggin (a potent antagonist of bone morphogenetic protein) was analyzed in schwannoma tissues and compared with that observed in other soft tissue tumors. Quantitative polymerase chain reaction analysis revealed that the mRNA levels of noggin in schwannomas were significantly increased compared with the levels in other tumors. The gene product of noggin was only detected in a subset of schwannomas using immunohistochemistry and western blot analysis. Furthermore, the tissue extract from a noggin-producing schwannoma was found to inhibit osteoblastic differentiation in MC3T3 mouse osteoblastic cells in a dose-dependent manner. These findings indicate that bone scalloping in radiology may be induced by schwannoma-secreted noggin. In addition, noggin may have potential as a novel molecular and diagnostic marker for identifying certain types of schwannoma.

## Introduction

Schwannomas, also known as neurilemmomas, are common, benign soft tissue tumors of nerve sheath origin. These slow-growing lesions arise from the peripheral, spinal or cranial nerves and commonly present several years prior to diagnosis (1–3). Characteristic bone scalloping of the spinal cord, including foramen enlargement, may develop when these tumors are located adjacent to a bone (4). Bone scalloping has been reported to occur in the bones of the extremities, as well as in vertebral bodies (5–8).

However, little has been reported on the periosteal reactions or sclerotic changes in the bones that are in contact with schwannomas. Bone scalloping is considered to be a radiologically benign indication of the prolonged existence of a tumor; however, the molecular mechanism underlying this process has yet to be elucidated.

To investigate the possible underlying mechanism of schwannoma-induced bone scalloping, it was hypothesized, in the present study that a specific extracellular factor, for example an inhibitor of bone formation, may be secreted from the tumor. Noggin, a potent antagonist of bone morphogenetic protein (BMP), inhibits BMP signal transduction through binding to ligands and consequently prevents the bone formation that is induced by BMP (9–12). In addition, noggin is expressed during the early development of the central nervous system (13) and has a major role in neural induction via the inhibition of BMPs (14–16). Although the expression of noggin in neurogenic tumors, including schwannomas, has yet to be investigated, this type of tumor may produce noggin given their neurogenic cellular origin.

In the present study the expression of noggin in soft tissue tumor samples, including schwannomas, was analyzed. The expression of noggin mRNA and protein was examined and the effect of the tissue extract from a noggin-producing schwannoma for BMP-induced osteoblastic differentiation *in vitro* was investigated. The present study proposes a possible pathomechanism of bone resorption by schwannomas.

## Materials and methods

### Tumor tissues

Tumor samples were obtained from the primary tumors of five patients with schwannoma and 30 patients with other soft tissue tumors (five hemangiomas, five lipomas, five malignant fibrous histiocytomas, five malignant schwannomas, five synovial sarcomas and five liposarcomas) at the Department of Orthopedic Surgery, Osaka University and the Osaka Medical Center for Cancer and Cardiovascular Diseases (Osaka, Japan). The histological diagnoses and subtypes were established via routine pathological evaluation according to the criteria, which followed the World Health Organisation classification system (17). Clinical data, including age, gender, location of the lesion and the radiological findings were obtained for the schwannoma samples. Written informed consent based on the Ethical Committees of Osaka University Graduate School of Medicine and the Osaka Medical Center for Cancer and Cardiovascular Diseases was obtained from each patient. The study was approved by the ethics committee of Osaka University (Suita, Japan).

### Reverse transcription (RT)-polymerase chain reaction (PCR) and quantitative (q)PCR

Tumor tissues were frozen immediately following surgical excision and stored at −80°C until the RNA extraction was performed. The total RNA was isolated using TRIzol^®^ Reagent (Invitrogen Life Technologies, Carlsbad, CA, USA) according to the manufacturer’s instructions. Complementary (c)DNA was generated using the Transcriptor First Strand cDNA Synthesis kit (Roche Diagnostics, Mannheim, Germany). The transcripts of noggin and the BMP antagonists*,* chordin and sclerostin, were analyzed in all of the tumor tissues. RT-PCR analysis was performed using a PCR Master Mix (Promega Corporation, Madison, WI, USA) with the following primer sequences: Forward, 5′-CTCGGGGGCCACTACGAC-3′ and reverse, 5′-GCACGAGCACTTGCACTCG-3′ for noggin; forward, 5′-AACACATGCTTCTTCGAGG-3′ and reverse, 5′-CTGTGGTTCCCAGAGGTAGTG-3′ for chordin; forward, 5′-CCGGAGCTGGAGAACAACAAG-3′ and reverse, 5′-GCACTGGCCGGAGCACACC-3′ for sclerostin; and forward, 5′-ACCACAGTCCATGCCATCAC-3′ and reverse, 5′-TCCACCACCTGTTGCTGTA-3′ for GAPDH. The PCR products were separated using agarose gel electrophoresis and detected using ethidium bromide. For the qPCR analysis, the expression of each mRNA was quantified using the LightCycler^®^ TaqMan^®^ Master kit (Roche Diagnostics). The Universal ProbeLibrary (UPL) probes used were as follows: Forward, 5′-GAAGCTGCGGAGGAAGTTAC-3′ and reverse, 5′-TACAGCACGGGGCAGAAT-3′ for noggin (UPL probe no. 5); and forward, 5′-AGACACATCGCTCAGACAC-3′ and reverse, 5′-GCCCAATACGACCAAATCC-3′ for GAPDH (UPL probe no. 60). The expression of noggin was normalized to that of GAPDH.

### Western blot analysis for noggin protein expression

The total protein extracted from the schwannoma samples was used for western blot analysis. Tumor tissue was homogenized in tissue protein extraction reagent buffer (Pierce Biotechnology, Inc., Rockford, IL, USA) containing a protease inhibitor cocktail (Thermo Fisher Scientific, Waltham, MA, USA) to avoid protein degradation and was solubilized using a 2× SDS-PAGE sample buffer. Samples were subjected to 4–12% SDS-PAGE and transferred onto nitrocellulose membranes (Bio-Rad Laboratories, Inc., Hercules, CA, USA). Subsequent to blocking with 0.1% Tween 20 in phosphate-buffered saline (PBS) containing 3% bovine serum albumin (BSA; Sigma-Aldrich, St. Louis, MO, USA) the membranes were incubated with specific rabbit polyclonal primary antibodies against noggin (ab16054; Abcam PLC, Cambridge, UK) or β-actin (Cell Signaling Technology, Inc., Beverly, MA, USA). Membranes were subsequently incubated with horseradish peroxidase-conjugated secondary antibodies (GE Healthcare, Little Chalfont, UK) and enhanced chemiluminescence reagents (GE Healthcare).

### Immunohistochemistry for noggin expression

Tissue sections were deparaffinized using xylene, dehydrated using graded alcohol and immersed in 70% methanol with H_2_O_2_ to block endogenous peroxidase activity. Antigen retrieval for noggin was performed using a microwave oven for 10 min in 10 mM citrate buffer (pH 7.0). Sections were incubated with 1% goat serum for 1 h at room temperature, washed in PBS and incubated with anti-noggin antibodies (ab16054) in 2% (w/v) BSA/PBS overnight at 4°C. Sections were washed three times with 0.1% (v/v) Tween 20/PBS followed by incubation and were analyzed using the EnVision™ system (Dako, Glostrup, Denmark). The staining intensity was scored according to the following scale: −, <10%; +, 10–45% positive cells; and ++, 46–95% positive cells.

### Effect of schwannoma tissue extract on the osteoblastic differentiation of MC3T3-E1 cells

Mouse preosteoblastic MC3T3-E1 cells were obtained from Riken Cell Bank (Tsukuba, Japan). The MC3T3-E1 cells were maintained in α-minimal essential medium (Invitrogen Life Technologies) and supplemented with 10% fetal bovine serum (Hyclone, Road Logan, UT, USA) in a humidified atmosphere of 5% CO_2_ at 37°C. For each assay, the growth medium was replaced with differentiation medium and supplemented with 0.2 mM ascorbic acid (Sigma-Aldrich) and 4 mM β-glycerophosphate (Sigma-Aldrich).

### Alkaline phosphatase (ALP) staining and activity in MC3T3-E1 cells

MC3T3-E1 cells were plated onto 24-well plates (Becton-Dickinson, Franklin Lakes, NJ, USA) at a density of 4×10^4^ cells/well. After 24 h, the cells were treated with various concentrations of homogenized schwannoma extract. The culture media was replaced with growth medium. Following three days of culture, cells were washed with PBS and fixed for 15 min with 10% formalin at room temperature. Following fixation, cells were incubated with the ProtoBlot AP System with Stabilized Substrate (Promega Corpration) for 1 h at room temperature. To measure ALP activity, the cells were washed with PBS and lysed in mammalian protein extraction reagent (Pierce Biotechnology, Inc.) according to the manufacturer’s instructions. ALP activity was measured using LabAssay™ ALP (Wako Pure Chemicals Industries, Ltd., Osaka, Japan) with p-Nitrophenyl phosphate as a substrate. To normalize the enzyme activity, the protein content was measured using a bicinchoninic acid protein assay kit (Pierce Biotechnology, Inc.).

### RT-PCR analysis for osteoblastic markers

The total RNA was isolated from the cells using TRIzol^®^ Reagent (Invitrogen Life Technologies) according to the manufacturer’s instructions. cDNA synthesis was performed using a cDNA synthesis kit (Roche Diagnostics) and RT-PCR analysis was performed using a PCR master mix (Promega Corporation) and the appropriate primer pairs. The specific primer sequences used for RT-PCR analysis were as follows: Forward, 5′-GCCCTCTCCAAGACATATA-3′ and reverse, 5′-CCATGATCACGTCGATATCC-3′ for ALP; forward, 5′-CAAGTCCCACACACAGCAGCTT-3′ and reverse, 5′-AAAGCCGAGCTGCCAGAGAGTT-3′ for osteocalcin; forward, 5′-GCAATCGGGATCAGTACGAA-3′ and reverse, 5′-CTTTCACGCCTTTGAAGCCA-3′ for collagen I; and forward, 5′-TGAACGGGAAGCTCACTGG-3′ and reverse, 5′-TCCACCACCCTGTTGCTGTA-3′ for GAPDH. The PCR products were separated using agarose gel electrophoresis and detected using ethidium bromide.

### Proliferation assay of MC3T3-E1 cells

MC3T3-E1 cells were cultured on 96-well plates (Becton-Dickinson) at a concentration of 2×10^4^/cm^2^. After three days of culture, cell proliferation was assessed using the Premix WST-1 cell proliferation assay system (Takara Bio, Inc., Otsu, Japan) according to the manufacturer’s instructions. This assay was performed every 24 h.

### Statistical analysis

All data are presented as the mean ± standard deviation and a minimum of three independent experiments were performed for each assay. Statistical analysis was performed using a two-sided unpaired Student’s t-test or analysis of variance for multiple comparisons. P<0.05 was considered to indicate a statistically significant difference.

## Results

### Detection of noggin mRNA in soft tissue tumors

**Figure 1:**
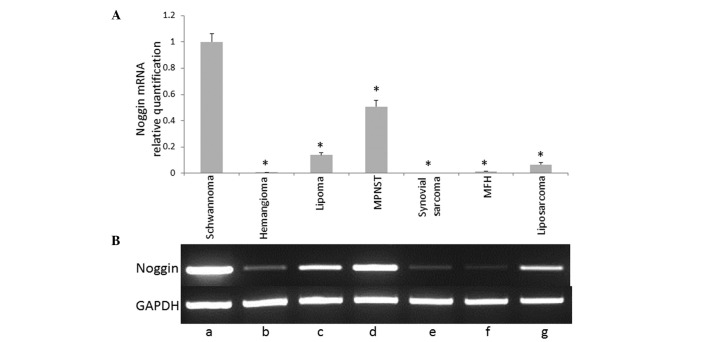
Noggin mRNA expression in soft tissue tumors. (A) Quantitative PCR analysis was performed on total RNA isolated from the schwannoma, hemangioma, lipoma, MPNST, synovial sarcoma, MFH and liposarcoma soft tissue tumors (n=5). Gene expression was normalized to GAPDH expression. Data are presented as expression relative to the control (schwannoma). Values are presented as the mean ± standard deviation. (B) Expression of noggin mRNA detected using reverse transcription PCR analysis. ^*^P<0.05 vs. the control. MPNST, malignant peripheral nerve sheath tumor; MFH, malignant fibrous histiocytoma; PCR, polymerase chain reaction.

**Figure 2:**
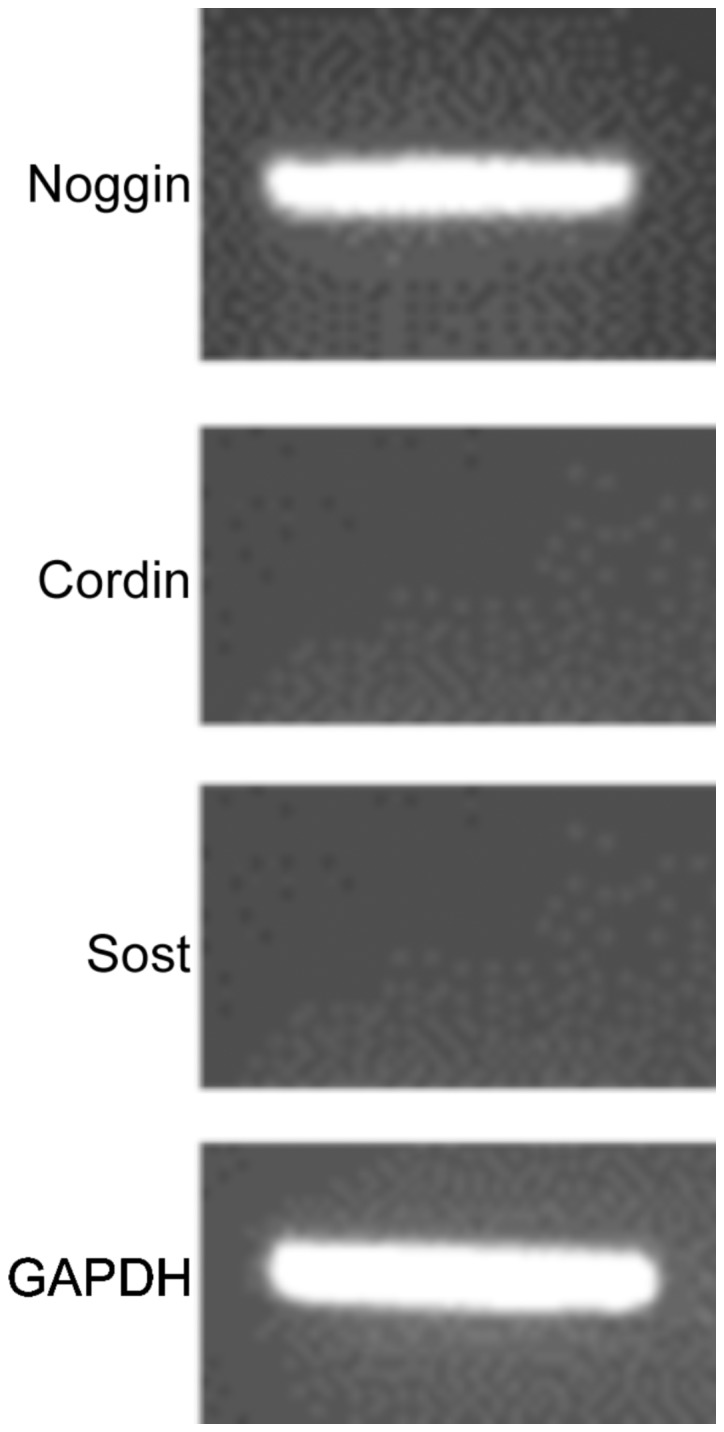
mRNA expression of bone morphogenetic protein antagonists in schwannoma using RT-PCR. Total RNA was isolated from schwannoma tissue and the expression of the cordin and Sost genes was assessed using RT-PCR. Sost, sclerostin; RT-PCR, reverse transcription-polymerase chain reaction.

Fig. 1 shows the mRNA expression profile of noggin in soft tissue tumors detected using RT-PCR analysis. Noggin mRNA expression was determined using qPCR analysis. Data are presented as relative quantification values against GAPDH. Noggin mRNA expression was found to be significantly increased in the schwannoma tissue compared with the other soft tissue tumors (P<0.05). The BMP antagonists, chordin and sclerostin, were not found to be expressed in schwannoma (Fig. 2) or other soft tissue tumors (data not shown).

### Noggin protein expression in soft tissue tumors

**Figure 3:**
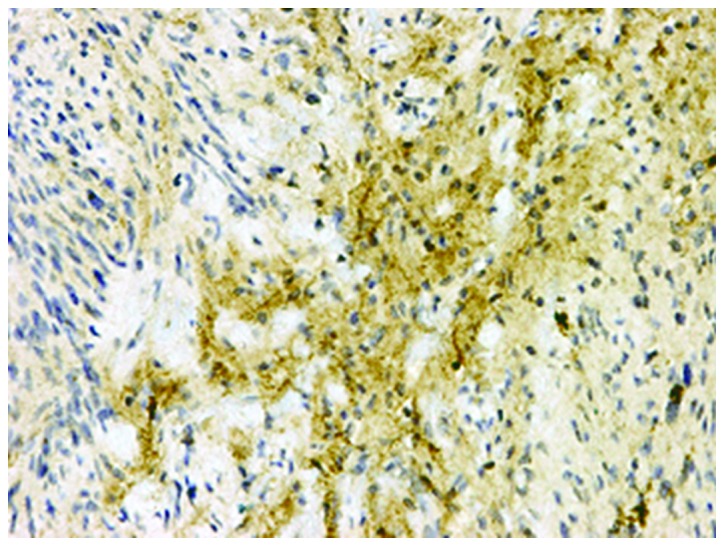
Noggin expression in a schwannoma tissue sample detected using immunohistochemistry. Immunostaining was localized to the cytoplasm of the spindle tumor cells (diaminobenzidine stain; magnification, ×200).

**Figure 4:**
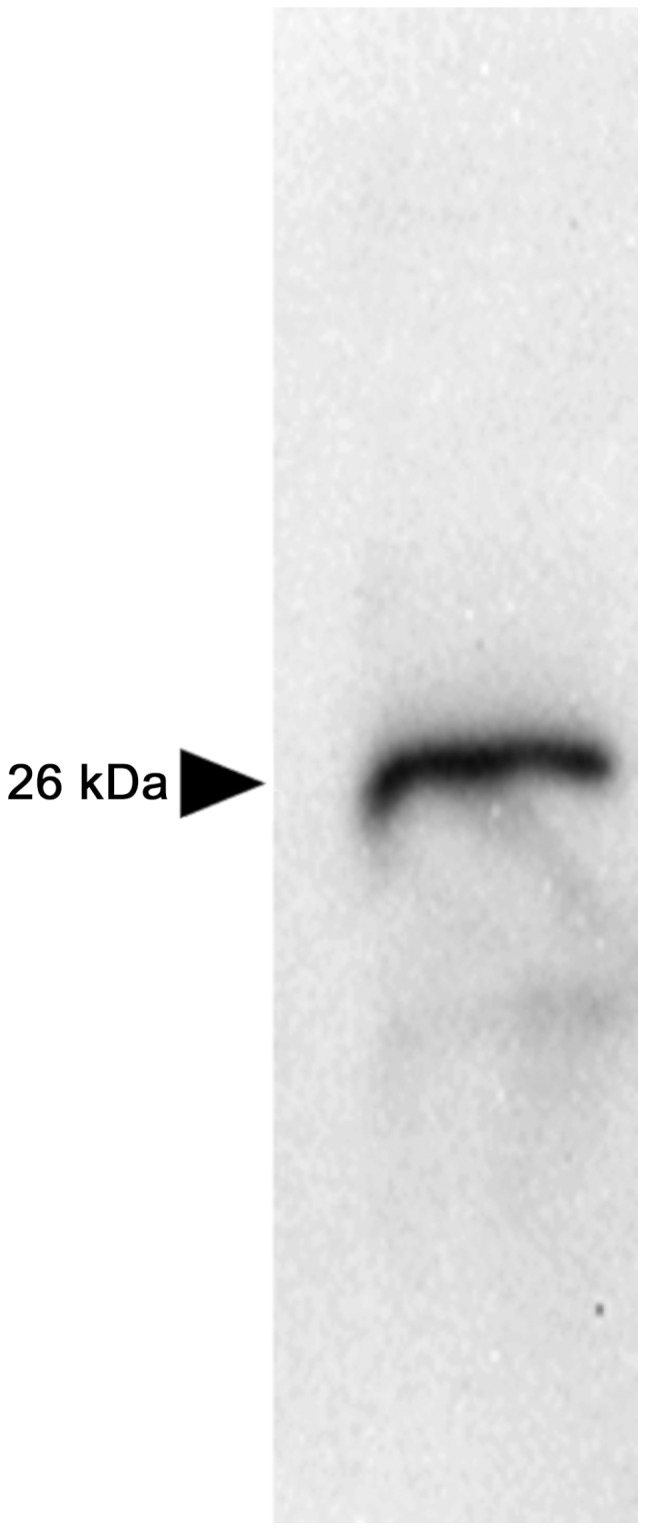
Western blot analysis of noggin protein expression in a schwannoma tissue. The total protein was extracted from a schwannoma tumor and was used for western blot analysis with anti-noggin antibodies. A single band of ~26 kDa was observed.

Table I shows the immunohistochemical analyses of the noggin protein in the soft tissue tumors. Noggin expression was detected in the schwannoma tissue, however, it was not detected in the other soft tissue tumors. In the schwannoma tissue samples, various levels of noggin immunoreactivity were observed in two of the five tissues. The immunostaining for noggin was localized to the cytoplasm of the spindle tumor cells, primarily demonstrating an Antoni B tissue pattern (Fig. 3). Western blot analysis in the schwannoma tissue revealed a single immunoreactive band corresponding with the size of the noggin protein, with a molecular mass of 26 kDa (Fig. 4).

### Effect of schwannoma tissue extract on the differentiation and proliferation of mouse MC3T3-E1 cells

**Figure 5:**
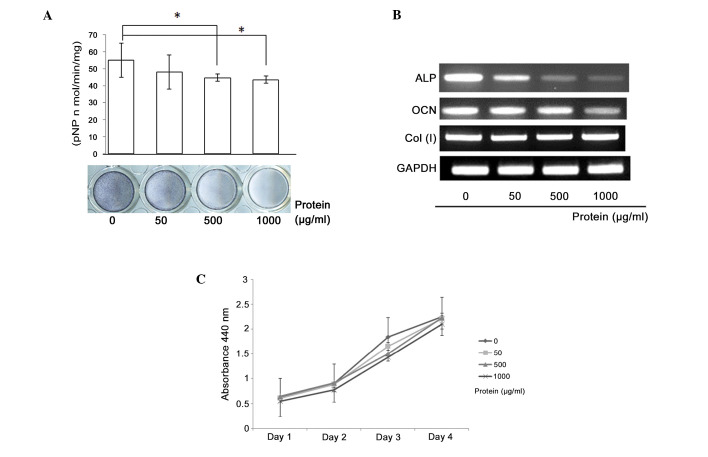
Effect of schwannoma tissue extract on the differentiation and proliferation of mouse MC3T3 osteoblasts. (A) ALP staining and activity in MC3T3 cells treated with the schwannoma tissue extract. Cells were cultured with various concentrations of the extract for three days and observed. (B) Reverse transcription-polymerase chain reaction analysis of total RNA isolated from MC3T3 cells treated with the schwannoma tissue extract, detecting the expression of osteoblastic associated genes, ALP, OCN and Col I. (C) Proliferation of MC3T3 cells treated with various concentrations of schwannoma tissue extract. Cells were incubated for a day following which the medium was treated with various concentrations of the extract for three days. Data are presented as the mean ± standard deviation of three independent experiments performed in duplicate. ^*^P<0.05 vs. the extract-untreated control. pNP, p-Nitrophenyl; ALP, alkaline phosphatase; OCN, osteocalcin; col, collagen.

Schwannoma tissue extracts containing the noggin protein were found to inhibit osteoblastic differentiation in MC3T3-E1 cells, resulting in a dose-dependent reduction in ALP activity (Fig. 5A). The ALP staining results correlated with the ALP activity results. RT-PCR analysis revealed a suppression of ALP and osteocalcin mRNA expression with increasing extract concentration (Fig. 5B). However, the proliferation of the MC3T3-E1 cells was not affected by the addition of the tissue extract (Fig. 5C).

### Clinical data of the patients with schwannoma and noggin expression patterns

Table II shows the clinical data of the patients with schwannomas and the noggin expression patterns in the schwannoma samples obtained from these patients. The tumor from case 1 was in contact with the bone and the patient exhibited typical bone scalloping. The tumors in the other cases were not in contact with the bone and no bone scalloping was observed. The immunoreactivity for noggin was positive in cases 1 and 2 and noggin mRNA was expressed in cases 1, 2, 3 and 4. Western blot analysis revealed that noggin protein expression was only detected in case 1.

## Discussion

Noggin, an extracellular homodimeric glycoprotein, is a bone morphogenetic protein antagonist, which binds to BMP-2/4 with high affinity; therefore, noggin interferes with BMP-receptor binding (18,19). Noggin is significant in the negative regulation of bone formation, including fracture healing (20,21). For example, a transgenic mouse overexpressing noggin exhibited decreased trabecular bone volume and osteopenia (9). In noggin-null mice, augmented BMP activity has been reported to evoke a series of developmental abnormalities, including dysmorphogenesis of the axial skeleton and joint lesions (10,22). Noggin was initially discovered due to its capacity to induce secondary axis formation in Xenopus embryos (15,23,24). Furthermore, the expression of noggin has been reported in the early development of the central nervous system, which indicates that noggin may be produced by neurogenic cells. Additionally, noggin regulates a BMP gradient-directed dorsal-ventral patterning with subsequent germ layer formation (25). However, it has also been reported that noggin is more widely expressed throughout the adult central nervous system and has been proposed to have an important role in the adult brain (26).

The present study detected the expression of noggin in schwannomas using RT-PCR analysis, immunohistochemistry and western blot analysis. Notably, the sample that exhibited vertebral bone scalloping also exhibited increased noggin mRNA and protein expression. Furthermore, RT-PCR analysis revealed that noggin mRNA levels were greatest in the schwannoma tissue and the second highest in the malignant neurogenic tumor tissue. These findings are in accordance with a previous report of noggin expression in the central nervous system (16).

In addition, in the present study, osteoblastic differentiation in MC3T3-E1 cells was found to be inhibited by schwannoma tissue extracts, which indicates that these extracts may include certain factors, which inhibit bone formation. As noggin is the most potent inhibitor of BMP, it may be the factor within the extract that is responsible for this inhibition.

Clinically, the most common imaging findings in spinal schwannoma include pedicle erosion, vertebral body scalloping and widening of the neural foramen (7,27–29). The pathomechanism of shwannnoma-induced foramen enlargement and vertebral scalloping has yet to be elucidated; however, it has been proposed that pressure erosion on the bone adjacent to the schwannoma may occur due to the gradual increase in schwannoma size (30,31). The findings of the present study indicate that schwannoma-derived noggin may induce a negative balance of bone remodeling via its BMP antagonist activity, resulting in local bone resorption.

In conclusion, the present study has detected the expression of noggin in schwannoma tissue samples. The analysis of noggin expression in a subset of schwannomas may provide a novel diagnostic tool for schwannoma. Noggin may be a useful molecular marker for the differential diagnosis of soft tissue tumors in pathology. Furthermore, the radiological bone scalloping and erosion observed in schwannoma patients may be caused by schwannoma-derived noggin.

## Figures and Tables

**Table I:** Immunohistochemical analyses of noggin in soft tissue tumors.

		Noggin expression			
		
Diagnosis	No.	++	+	−	Rate (%)
Schwannoma	5	1	1	3	40
Hemangioma	5	0	0	5	0
Lipoma	5	0	0	5	0
MPNST	5	0	0	5	0
MFH	5	0	0	5	0
Synovial sarcoma	5	0	0	5	0
Liposarcoma	5	0	0	5	0

++, 46–95% positive cells; +, 10–45% positive cells; −, <10% positive cells. MPNST, malignant peripheral nerve sheath tumor; MFH, malignant fibrous histiocytoma.

**Table II:** Clinical data of patients with schwannoma and expression patterns of noggin in the tumors.

					Expression of noggin		
					
No.	Gender/Age	Location	Contact with bone	Bone scalloping	IHC	RT-PCR	Western blot analysis
1	F/59	Spine	(+)	(+)	(++)	(+)	(+)
2	F/63	Spine	(−)	(−)	(+)	(+)	(+)
3	F/49	Supraclavicular fossa	(−)	(−)	(−)	(+)	(−)
4	F/31	Elbow	(−)	(−)	(−)	(+)	(−)
5	F/51	Foot	(−)	(−)	(−)	(−)	(−)

IHC, immunohistochemistry; RT-PCR, reverse transcription polymerase chain reaction; F, female; +, expression; −, no expression.
